# Antiangiogenic activity of photobiomodulation in experimental model using chorioallantoic embryonic membrane of chicken eggs

**DOI:** 10.5935/0004-2749.2021-0524

**Published:** 2022-09-06

**Authors:** Lays Fernanda Nunes Dourado, Rubens Camargo Siqueira, Ana Paula Alves, Mayara Rodrigues Brandão de Paiva, Ubirajara Agero, Armando da Silva Cunha Junior

**Affiliations:** 1 Faculty of Pharmacy, Universidade Federal de Minas Gerais, Belo Horizonte, MG, Brazil; 2 Faculdade de Medicina de São José do Rio Preto, São José do Rio Preto, SP, Brazil; 3 Institute of Exact Sciences, Physics Department, Universidade Federal de Minas Gerais, Belo Horizonte, MG, Brazil

**Keywords:** Photobiomodulation, Chorioallantoic membrane, Red light therapy, Angiogenesis, Age-related macular degeneration, Retinal vessels, Fotobiomodulação, Membrana corioalantoica, Te rapia com luz vermelha, Angiogênese, Degeneração macular relacionada à idade, Vasos retinais

## Abstract

**Purpose:**

The purpose of this study was to investigate the vascular effects of photobiomodulation using a light-emitting diode on the chorioallantoic embryonic membrane of chicken eggs grouped into different times of exposure and to detect the morphological changes induced by the light on the vascular network architecture using quantitative metrics.

**Methods:**

We used a phototherapy device with light-emitting diode (670 nm wavelength) as the source of photobiomodulation. We applied the red light at a distance of 2.5 cm to the surface of the chorioallantoic embryonic membrane of chicken eggs in 2, 4, or 8 sessions for 90 s and analyzed the vascular network architecture using *AngioTool* software (National Cancer Institute, USA). We treated the negative control group with 50 µl phosphate-buffered-saline (pH 7.4) and the positive control group (Beva) with 50 µl bevacizumab solution (Avastin, Produtos Roche Químicos e Farmacêuticos, S.A., Brazil).

**Results:**

We found a decrease in total vessel length in the Beva group (24.96% ± 12.85%) and in all the groups that received 670 nm red light therapy (2× group, 34.66% ± 8.66%; 4× group, 42.42% ± 5.26%; 8× group, 38.48% ± 6.96%), compared with the negative control group. The fluence of 5.4 J/cm^2^ in 4 sessions (4×) showed more regular vessels. The number of junctions in the groups that received a higher incidence of 670 nm red light (4× and 8×) significantly decreased (p<0.0001).

**Conclusion:**

Photo-biomodulation helps reduce vascularization in chorioallantoic embryonic membrane of chicken eggs and changes in the network architecture. Our results open the possibility of future clinical studies on using this therapy in patients with retinal diseases with neovascular components, especially age-related macular degeneration.

## Abstract (pt)

**Objetivo:**

investigar os efeitos vasculares da foto-biomodulação com diodo emissor de luz utilizando membrana embrionária corioalantóide de ovos de galinhas em grupos com diferentes tempos de exposição e detectar as alterações morfológicas por meio de métricas quantitativas promovidas pela luz na arquitetura da rede vascular.

**Métodos:**

Um aparelho de fototerapia com diodo emissor de luz no comprimento de onda de 670 nm foi usado como fonte de fotobiomodulação. A luz vermelha foi aplicada a uma distância de 2,5 cm da superfície da membrana embrionária corioalantóide em 2, 4 ou 8 sessões de 90 s a arquitetura da rede vascular foi analisada por meio do software *AngioTool* (National Cancer Institute, USA). Usamos um grupo controle negativo tratado com 50 µL de solução salina tamponada com fosfato (PBS) pH 7,4 e um grupo controle positivo (Beva) tratado com 50 µL de solução de bevacizumabe (Avastin, Produtos Roche Químicos e Farmacêuticos S.A., Brasil).

**Resultados:**

Uma diminuição no comprimento total do vaso foi detectada para o grupo Beva (24,96 ± 12,85%), e para todos os grupos que receberam terapia de luz vermelha de 670 nm, 34,66 ± 8,66% (2x), 42,42 ± 5,26% (4x) e 38,48 ± 6,96% (8x) em comparação ao grupo controle. A incidência de 5,4 J/cm^2^ em 4 sessões (4x) mostrou vasos mais regulares. A redução foi mais intensa nos grupos que receberam maior incidência de luz vermelha de 670 nm (4x e 8x).

**Conclusão:**

A fotobiomodulação contribui para a redução da vascularização nos vasos da membrana embrionária corioalantóide de ovos de galinhas e mudanças na arquitetura da rede. Os achados deste experimento abrem a possibilidade de considerar um estudo clínico usando esta terapia em pacientes com doenças retinais com componentes neovasculares, especialmente degeneração macular relacionada à idade.

## INTRODUCTION

Photobiomodulation therapy uses red or near-infrared (NIR) light with wavelength from 600 to 1000 nm. Previous research on its mechanism usually demonstrated that red or NIR light preserves and restores cellular functions by reversing the dysfunctional mitochondrial cytochrome C oxidase (COX) activity^(1,2)^.

The COX, a terminal electron acceptor in the mitochondrial electron transport chain, is an important photoacceptor molecule^(3)^. When activated by red or NIR light, it drives intracellular changes such as metabolic rate increase, cell migration, cell proliferation, and protein secretion^(4,5)^. A previous study demonstrated that mitochondrial function shifts after selective absorption of red light, leading to increased ATP production^(6)^.

Recently, the number of studies on the use of photobiomodulation on ocular diseases has grown. Ocular effects promoted by ocular photobiomodulation can be predicted on the basis of the mechanisms of this therapy on other tissues^(7,8)^.

The Food and Drug Administration recently approved the use of photobiomodulation therapy, which showed an effect on age-related macular degeneration (AMD), for clinical studies ^(8,9)^. Although most studies demonstrated the benefits of red light or NIR treatment on experimentally induced retinal damage, the treatment seems to have more activity in the early stages of the disease in clinical practice.

Most studies investigating the efficacy of photobio-modulation used light-induced retinal damage because light induces tissue damage similar to the pathology of AMD^(10,11)^, a common cause of blindness worldwide. A previous study estimated that around 196 million people have AMD and that the prevalence rate will increase to 288 million in 2040^(12)^.

The multifactorial etiology of AMD includes factors such as aging, dietary habits, cigarette smoking, and phototoxic exposure, which can trigger inflammation and tissue stress leading to the initiation and/or progression of AMD. In addition, these can also lead to more severe complications of AMD, (i.e., choroidal neovascularization [CNV])^(13)^.

The CNV process is characterized by the formation of new vessels that are fragile and break easily, leading to blood leakage in the retina and sudden vision loss^(13,14)^. Therefore, neovascularization is a potential therapeutic target for the treatment of retinal degeneration diseases^(15)^. However, in models of light-induced retina damage, the CNV process cannot be evaluated.

On the other hand, numerous studies used the chorioallantoic embryonic membrane (CAM) of chicken eggs to evaluate the antiangiogenic activity of drugs^(16,17)^. Moreover, others used the CAM to evaluate toxicity^(18)^, ocular irritation^(16,19)^, antitumor activity^(20)^, and biocompatibility^(21,22)^.

The CAM, an extraembryonic membrane that mediates gas and nutrient exchanges and a vascular network connected to the embryonic circulation via the allantoic arteries, appears around 3.5 days after incubation. Between days 4 and 8, junctions appear and become capillaries. This rapid capillary proliferation continues until day 11, followed by a rapid decline in the mitotic index until hatching on day 18^(22,23)^.

This study aimed to investigate the red light effect on CAM vessels in groups with different times of exposure and to detect the morphological changes induced by the light on the vascular network architecture using quantitative metrics.

## METHODS

### Evaluation of the antiangiogenic activity of the 670 nm red light exposure on the CAM model

We obtained fertilized chicken eggs (*Gallus gallus dosmeticus*) from a local hatchery (Alimentos Rivelli, Brazil) and incubated them at 37°C ± 5°C and 60% ± 2% relative humidity. On day 3 of embryo development, we opened a 2 cm^2^ hole in the eggshell air chamber and exposed the CAM. We then sealed the hole using sterile adhesive tape, and placed the egg back to the incubator. On days 5 and 6, we applied light with a wavelength (λ infrared) of 670 nm using a device (Quantum Devices, USA) at a distance of 2.5 cm to the CAM surface in 2, 4, or 8 sessions for 90 s (n=6). Each turn corresponded to an irradiance intensity of 60 mW/cm^2^, delivering 5.4 J/cm^2^ per treatment (i.e., 2× = 10.8 J/cm^2^; 4× = 21.6 J/cm^2^ and 8× = 43.2 J/cm^2^).

To compare the influence of 670 nm red light on vessel growth, we used negative and positive (Beva) control groups treated with 50 µL of phosphate-buffered-saline (PBS, pH 7.4) and 50 µL of bevacizumab solution (treated on day 4 of embryonic development; Avastin, Produtos Roche Químicos e Farmacêuticos, S.A., Brazil), respectively, at 250 µg/ml, a monoclonal antibody against vascular endothelial growth factor. We did not apply light on the negative and positive controls.

On day 8, we photographed the CAM of each group using a stereomicroscope (Leica, model DM4000B, Germany). We combined a digital CCD camera (model DFC 280) with Leica and obtained the original images using its software (Leica Application Suite V 3.3.0, Germany). We then processed the microphotographs using *ImageJ* software (version 1.50i; National Institutes of Health, USA).

**Figure 1:**
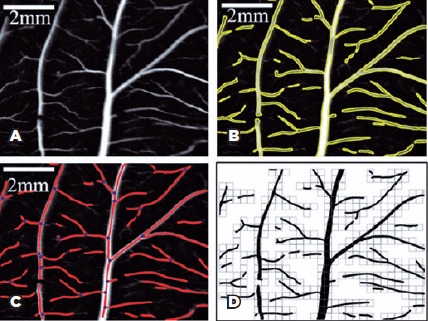
Detection of vascular network architecture. (A) A typical image processed on *ImageJ* software. (B) The vascular area identifed on *AngioTool* software. (C) The vascular network architecture processed on *AngioTool* software, displaying the junctions that represent the vessel bifurcations and the links identifed by the vessel branches. (D) A typical binary vascular network architecture used to calculate the fractal dimension using *FracLac* software, displaying the box size of 32 pixels.

We converted the images to grayscale. Figure 1A shows a typical image obtained from CAM in this process. We then analyzed the images using *AngioTool* software (National Cancer Institute, USA). We detected the vessel area by selecting a threshold of 26 for the gray level in the software; the mean value for the selected vessel thickness was close to 0.05 ± 0.01 mm. Figure 1B shows a typical image displaying the vessel.

The *AngioTool* software identified the vessel branches and quantified the vessel area, vessel length, and the number of junctions on the network architecture^(24)^. After processing the image in the *AngioTool* software, we determined the network architecture as shown in figure 1C. The junctions on the network architecture represented the vessel bifurcation, whereas the links among them are the branches. Figure 1D shows a typical image displaying the binary vascular network architecture.

To measure the spatial branch distribution in the vascularized area, we measured the fractal dimension (D_f_) using the box-counting method on the binary branch patterns, with box sizes ranging from 10 pixels to 700 pixels, following a power series. We used the D_f_ to study the homogeneity of several systems in nature^(25^, ^26^, ^27)^, including vessel branches^(28,29)^. In this case, the D_f_ measures the space-filling using the branches in the vascular network under different treatments; since the D_f_ will be larger than that of a line but smaller than that of a plane, it will be in the interval between one and two (1 < D_f_ < 2). To calculate D_f_, we used *FracLac software*^(30)^ in ImageJ software. A smaller D_f_ indicates that the system displays few branches on the analyzed area. On the other hand, a higher D_f_ indicates that the spatial pattern of the vascular network is filled with vessel branches. Another metric that we used to analyze the homogeneity of spatial patterns is the lacunarity, which has been widely applied to analyze vascular branch patterns ^(24,26,27)^.

Lacunarity measures the gap in the spatial distribution of branches in the vascular network architecture: a low lacunarity value indicates that the spatial pattern displays a homogeneous branch distribution, whereas a high lacunarity value indicates a heterogeneous branch distribution.

### Statistical analysis

We performed the statistical analysis and creation of graphs using GraphPad Prism software (California, USA version 8.4.2 for Windows). We set the criterion for significance at p-value <0.05 for all comparisons and expressed the results as mean ± standard error of the mean (SEM). We fixed the negative control group at 100% and analyzed the comparison among groups using one-way ANOVA followed by Tukey’s.

## RESULTS

### Evaluation of the antiangiogenic activity of 670 nm red light therapy in CAM

**Figure 2:**
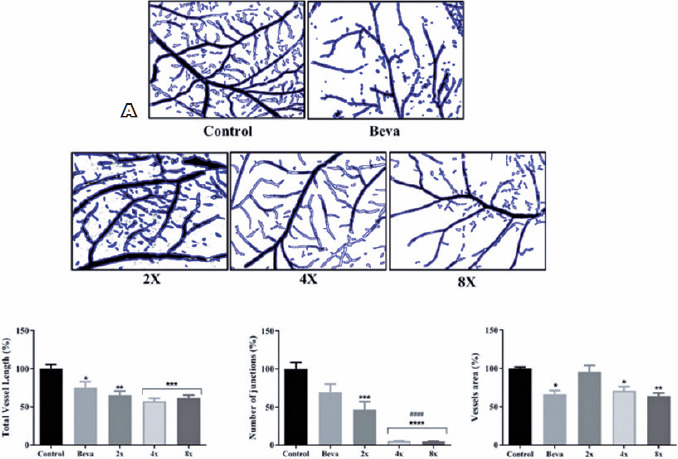
Red light induces changes on the vessel distribution. The vascularized area is identifed using *AngioTool* software and is outlined by the blue line. (A) The quantification of angiogenic responses for total vessel length, number of junctions, and vessel area is represented in the three graphs at the bottom of the figure. The negative control group was fixed as 100%. The statistic was performed using one-way ANOVA, followed by Tukey test. Data represent the means ± SEM (*n*=6). Abbreviation: SEM, standard error of the mean. *p*-Value <.05. * indicates a difference with a *p*-value <.05 versus the negative control group. ** indicates a difference with a *p*-value <.01 versus the negative control group. *** indicates a difference with a *p*-value <.001 versus the negative control group. **** indicates a difference with a *p*-value <.0001 versus the negative control group. ^####^ indicates a difference with a *p*-value <.0001 versus the Beva group.

In the CAM assay, we measured the vessel distribution after exposure to 670 nm red light with different exposure times. Figure 2A shows the vascularized area identified by *AngioTool* for the control groups and treated groups, while Figure 2B shows the collected data on total vessel length, the number of junctions, and the vascularized areas.

**Figure 3:**
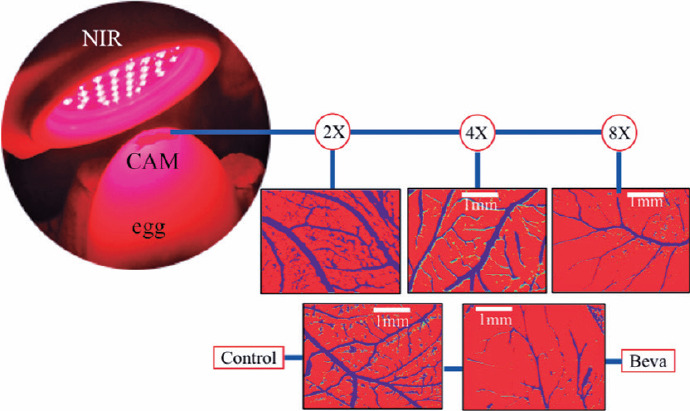
The upper part of the figure shows a sequence of 2, 4, and 8 applications of PBM and the progressive decrease of the vascular network similar to the action of bevacizumab (below).

We found a decrease in total vessel length in the Beva group (24.96% ± 12.85%) and in all groups that received 670 nm red light therapy (2× group, 34.66% ± 8.66%; 4× group, 42.42% ± 5.26%; and 8× group, 38.48% ± 6.96%) compared with the negative control group. Similarly, the number of junctions decreased in the Beva group (30.40% ± 19.20%) and in all light-treated groups (2× group, 53.43% ± 19.40%; 4× group, 94.87% ± 0.74%; and 8× group, 95.37% ± 0.43%). The groups that received a higher incidence of 670 nm red light (4**×** and 8**×**) had more significant decreases. The vessel area displayed a behavior resulting from changes in the vessel length and the number of junctions. The Beva group showed a reduction of 27.84% ± 12.26%, similar to the 4× group (29.23% ± 10.21%). The 8× group had more significant reduction (36.02% ± 6.03%). On the other hand, the 2× group did not show a significant vessel area reduction (4.31% ± 12.14%) (Figure 3).

**Figure 4:**
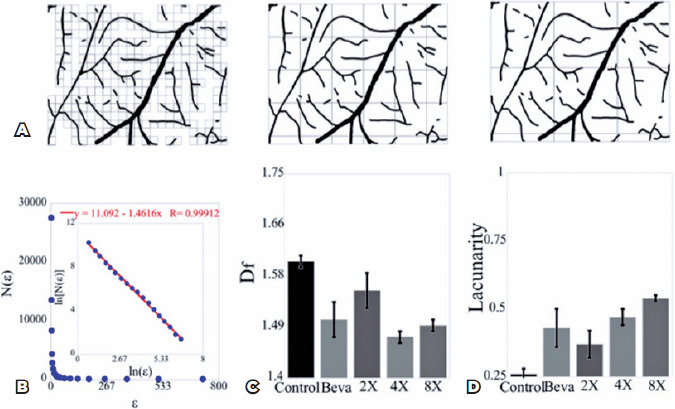
Red light induces changes in the binary vascular network pattern. (A) Several box sizes or scales used to scan the binary vascularized area. (B) Data on the total number of box size, N (Ɛ), as a function of the scale, Ɛ, in pixels used to scan the vascularized area. The ft is obtained using the linear function where the fractal dimension represents a negative slope of the ft (D_f_=1.46), and the R^2^ coefficient has three nines (R=.999). (C) The mean value for the fractal dimension was obtained for all groups. (D) The mean value for the lacunarity was obtained for all groups. The error bars represent the means ± SEM from six samples in each group. Abbreviation: SEM, standard error of the mean.

The D_f_ measurements showed homogeneous spatial branch distribution in the analyzed area, as in Figure 4A. The D_f_ is proportional to the number of boxes required to fill all the branch distribution, N(Ɛ), where Ɛ represents the box size in pixels used to scan all the branches in the analyzed area. The relationship between the number of boxes, the scale, and the D_f_ is mathematically expressed as N(Ɛ) = Ɛ^-Df^. Figure 4B shows a typical data distribution for the number of boxes, N(Ɛ), as a function of the scale, Ɛ.

To obtain the D_f_, we obtained the plot of the ln[N(Ɛ)] as a function of ln(Ɛ) and fit the data using a linear function. The negative slope of the fit represents the D_f_ at 1.46 (Figure 4B). To quantify the changes in the spatial patterns, we exposed the vascular network architectures to different 670 nm red light exposure treatments. As shown in Figure 4C and 4D, we measured the mean D_f_, and the mean lacunarity from the binary vascular network architectures. We also obtained the mean D_f_ for all CAM samples. The value obtained in the negative control group (D_f control_) was 1.60 ± 0.01. On the other hand, the D_f_ obtained in the Beva group (D_f Beva_) was 1.50 ± 0.03, which is close to the D_f_ in the 8× group (D_f 8×_ = 1.49 ± 0.01), as shown in Figure 4C. We obtained a higher D_f_ in the negative control group and the smallest fractal in the groups that were exposed to the 670 nm red light longer. These suggest that light exposure decreases the space-filling of the branches in the vascular network architecture.

To measure spatial homogeneity in the vascular patterns, we measured the lacunarity, a metric widely used to investigate the spatial distribution of the vascular network^(24,26,27)^. The lacunarity measures the empty spaces in the spatial distribution. Figure 4D shows the mean lacunarity value for all CAM samples. The mean value measured for the negative control group (L_control_) was 0.26 ± 0.02, the lowest lacunarity value measured among all groups, indicating that the branch distribution in the negative control group displayed the most homogeneous spatial distribution. On the other hand, the lacunarity measured for the 8× group was the highest among all groups (L _8×_ = 0.54 ± 0.10), which is close to the value of the Beva group (L_Beva_ = 0.43 ± 0.07).

High lacunarity values indicate that the spatial distribution displayed heterogeneous branch patterns or that the empty spaces in these groups were more frequent.

## DISCUSSION

The CAM assay has been proposed as an alternative test to study angiogenesis in response to stimulus or inhibitor factors as it does not require ethics committee approval for animal experimentation because the chick embryo is unable to experience pain until day 15 of its development^(20^, ^21,22)^. The CAM assay represents an improvement in evaluating the photobiomodulation protocols and allows the understanding of the effects on blood vessels. Furthermore, the reasonable use of this method can potentially refine animal experimentation and can support a considerable reduction and/or replacement of animal experiments following the Principles of Humane Experimental Technique^(7,21,22)^.

In some aspects, we can note the similarity between the network of CAM vessels and the network in the posterior segment of the human eye^(28)^. Therefore, the CAM assay can be used as an important tool to investigate the ocular toxicity of different substances, especially drugs, and to evaluate different photosensitizer agents with potential application in photodynamic therapy. In addition, it is noteworthy that the CAM assay can act as a bridge between in vitro and in vivo experiments^(29,30)^.

We investigated the effect of red light, used in light therapy for retinal diseases, on CAM vessels. In this study, red light-induced a reduction in vessel length and junction number, contributing to a lower vascularized area, as supported by the lacunarity results. We found that the negative control group had the lowest lacunarity value. Therefore, the negative control group’s vascular network architecture displayed a more spatial homogeneous distribution. On the order hand, we found a higher lacunarity value in the groups treated with red light and the Beva group, suggesting that the gaps filled by the branches in the vascular network architecture in these groups are more frequent. Bevacizumab, which was used as a positive control in this work, has been used as a potent antiangiogenic drug for ocular neovas-cularization diseases^(27)^. The quantitative metrics used in this study strongly suggest that red light caused an antiangiogenic effect in vessel formation, mainly in the 4× and 8× groups.

Red light can also induce endothelium damage, leading to increased vascular permeability and inflammation under intensive conditions^(29)^. This process stimulates the production of angiogenic factors, such as VEGF, resulting in angiogenesis with new vessel formation replacing the original vessels^(28,29)^. The architecture of the newly formed vessels seems more tortuous and less organized compared with the original capillary plexus. In these vessels, the blood flows slowly and inefficiently, sharply compromising blood perfusion^(30)^.

To investigate the vascular network architecture, we used the D_f_ as it was used by Jurczyszyn and collaborators^(31)^. The Beva and red light-treated groups showed a low D_f_ value compared with the negative control group, reaffirming that red light therapy leads to a less chaotic blood vessel arrangement. The 4× group had the lowest value, suggesting that exposure (4 sessions of 90 s each) can reduce the vessel area, promoting more regular vessels.

Previous studies have demonstrated the benefits of exposure to red light-emitting diode in various retinal disease models, including light-induced retinal degeneration^(7,21,22)^. However, its mechanism has remained not completely understood.

On the basis of our results, we could infer that treatment with a fluence of 5.4 J/cm^2^ in 4 sessions can reduce new vessel formations, suggesting its use for neovascular diseases, such as AMD. Future experiments are needed to confirm this effect and to verify the efficacy in humans.

In conclusion, our results showed that red light therapy helps reduce vascularization in CAM vessels and changes in the network architecture. The results highlight the potential of CAM assay in understanding red light therapy effects on blood vessels and optimizing the protocols of treatments, thereby opening the possibility future clinical studies on the use of this therapy in patients with neovascular AMD.
